# Effects of Isalo scorpion cytotoxic peptide on growth, immune performance, and intestinal flora of yellow-feathered broilers

**DOI:** 10.3389/fvets.2024.1511680

**Published:** 2024-12-18

**Authors:** Zhengli Wang, Jianjun Zhu, Yan Ma, Tingting Liu, Shuaihu Chen, Mingyang Gao, Sijia Wang, Jungang Wang, Hong Shen

**Affiliations:** Shihezi University, Collage of Animal Science & Technology, Xinjiang, China

**Keywords:** antimicrobial peptide, ISCT, broilers, growth, immune, gut microbiota

## Abstract

**Introduction:**

This study aimed to investigate the effects of Isalo scorpion cytotoxic peptide (IsCT) on the growth performance, immune function, and gut microbiota of yellow-feathered broilers.

**Methods:**

The experiment involved supplementing a corn-soybean meal-based diet with various dietary levels of IsCT. The experiment adopted a completely randomized design. A total of 240 one-day-old yellow-feathered broilers were randomly divided into six groups: a control group (CON), a chlortetracycline group (CTC), and four groups receiving a basal diet with 50, 100, 150, or 200 mg/ kg IsCT (IsCT50, IsCT100, IsCT150, and IsCT200). The trial period lasted 60 days.

**Results:**

The results showed that the average final body weight and average daily weight gain of the IsCT150 and IsCT200 groups were significantly higher than those of the CON group (*p* < 0.05). Compared to the CTC group, the average daily feed intake in group III was significantly lower (*p* < 0.05). The feed conversion ratios of the IsCT150 and IsCT200 groups were significantly lower than those of the IsCT50 and CON groups (*p* < 0.05). Albumin levels in the CTC and IsCT150 groups were significantly higher than those in the CON, IsCT100, and IsCT50 groups. Alanine transaminase levels in the IsCT150 group were significantly lower than those in the IsCT50, IsCT200, CON, and CTC groups (*p* < 0.05), but there was no significant difference compared with the IsCT100 group (*p* < 0.05). IgA levels in the IsCT150, IsCT200, and CTC groups were significantly higher than those in the IsCT50 group (*p* < 0.05). IgG levels in the CTC, IsCT100, IsCT150, and IsCT200 groups were significantly higher than those in the IsCT50 and CON groups (*p* < 0.05). 16S rRNA sequencing indicated significant differences in the caecal microbiota between the IsCT and CON groups (*p* < 0.05). The abundance of *Bacteroidetes* increased, whereas that of Firmicutes decreased in the IsCT groups; however, the difference was not significant (*p* < 0.05). The relative abundances of *Actinobacteriota* and *Patescibacteria* were significantly different in the IsCT100 group (*p* < 0.05).

**Discussion:**

In conclusion, supplementing the basal diet with IsCT improved growth performance, immune function, and gut microbiota of yellow-feathered broilers at an optimal supplementation level of 150 mg/kg.

## 1 Introduction

The poultry industry plays a crucial role in ensuring global food security and nutritional supply by providing high-quality protein in the form of affordable and nutritious chicken meat and eggs (1). Global *per capita* meat consumption is increasing, with poultry accounting for 70% of the total meat consumption; over 66 billion broilers are slaughtered worldwide annually (2). Antibiotics have been used for poultry farming since the late 1940s. Consequently, the widespread use of antibiotics as feed additives has become a common practice (3). The extensive use of antibiotics has resulted in the accumulation of antibiotic-resistant bacteria with selection of antibiotic resistance genes in the environment, posing potential risks to ecosystems and human health (4, 5). The intake of antibiotics by food-producing animals and the resulting antibiotic residues in food have been recognized as major causes of the rapid spread of antimicrobial resistance in humans (6, 7). The continuous intake of antibiotics can result in an imbalance in the gut microbiota (8) and induce several intestinal diseases (9, 10). The 2019 Global Burden of Disease Report estimated that 14 million people died from infection-related causes, making it the second leading cause of death after ischaemic heart disease, with bacterial pathogens responsible for 7.7 million deaths, and antibiotic resistance in bacteria causing 1.3 million deaths (11). Therefore, there is an urgent need to develop green, natural, and non-toxic feed additives.

Antimicrobial peptides (AMPs) are small peptides produced by the innate immune system and are characterized by structural diversity and broad-spectrum antimicrobial activity (12, 13). As dietary additives, AMPs can improve broiler growth performance, immune organ indices, and gut microbiota (14–16). The bactericidal capacity of AMPs generally depends on their ability to interact with microbial cell membranes and cell walls. Most AMPs selectively bind to negatively charged cell membranes/cell walls owing to their highly positive charge and hydrophobic primary structure, resulting in non-enzymatic destruction of the cell membrane, thus making it difficult for bacteria to develop resistance (17–19). For example, the antimicrobial peptides magainin 2 and PGLa are synergistically inserted into the phospholipid bilayer to form pores that inactivate microorganisms (20). Some peptides can enter the cell without disrupting the cell membrane and kill bacteria by inhibiting crucial intracellular functions (21). Antimicrobial peptides such as cathelicidins can induce the production of intracellular reactive oxygen species (ROS), thereby damaging bacterial molecules and leading to slow growth or cell death (22). The antimicrobial peptide Mastoparan X reduced the serum levels of inflammation-related genes IL-6 and LITNF in broilers, while increasing the mRNA expression levels of the antioxidant genes CAT, HMOX1, and SOD1 (23). Sublancin, an antimicrobial peptide, helps alleviate necrotizing enteritis induced by *Clostridium perfringens* (24).

The Isalo scorpion cytotoxic peptide (IsCT) (25) is a short natural cytotoxic peptide. IsCT has demonstrated good *in vitro* antimicrobial activity against both Gram-negative and Gram-positive bacteria (26); moreover, it has shown significant antimicrobial effects against multidrug-resistant pathogens, including methicillin-resistant *Staphylococcus aureus* (MRSA) and other pathogens (27). Studies have indicated that IsCT improves gut barrier function, immune function, and gill health in grass carps (28–30).

This study aimed to explore the effects of different doses of IsCT supplementation in the diet of yellow-feathered broilers on their growth performance, serum immune indicators, and immune organ indices. Moreover, using 16S rDNA high-throughput sequencing analysis of the caecal microbiota, we investigated the effects of IsCT on the gut microbiota of yellow-feathered broilers, thus providing a theoretical basis for the application of antimicrobial peptides as novel feed additives in livestock and poultry farming.

## 2 Materials and methods

### 2.1 Materials and reagents

The antimicrobial peptide used in this experiment (scorpion-derived antimicrobial peptide IsCT) was prepared by solid-phase synthesis and was provided by Shenzhen SunSmile Biotechnology Co., Ltd. with a purity of 95%.

### 2.2 Animal rearing and experiments

This study was approved by the Animal Ethics Committee of the College of Animal Science and Technology at Shihezi University (A2024-14). The feeding trials were conducted at the Animal Experiment Station of the College of Animal Science and Technology, Shihezi University. Animal experiments lasted 60 days. A total of 240 healthy yellow-feathered broiler chickens, 1-day-old, with equal numbers of males and females, and an average body weight of (30 ± 1.4) g, were randomly divided into 6 groups, with 4 replicates per group and 10 chickens per replicate. Broiler chickens were reared according to the Technical Regulations for the Rearing Management of Yellow-Feathered Broiler Chickens in China (NY/T 1871–2010). AMP was added at four levels (50, 100, 150, and 200 mg/kg based on the dry matter weight of the diet) and the antibiotic control (CTC) group had an additive level of 75 mg/kg (based on the dry matter weight of the diet). The experimental design is summarized in Table 1. The basal diet was formulated according to the nutritional requirements of medium-speed yellow-feathered broiler chickens in NY/T 3645–202, and its composition is shown in Table 2. Both the basal and experimental diets were prepared using a total mixed ration blending mechanism. The dietary IsCT inclusion level was determined based on preliminary experiments (unpublished data).

**Table 1:** Experimental design.

Groups	Experimental treatment
CON	Basal diet
CTC	Basal diet +75 mg/kg Aureomycin
IsCT50	Basal diet +50 mg/kg IsCT
IsCT100	Basal diet +100 mg/kg IsCT
IsCT150	Basal diet +150 mg/kg IsCT
IsCT200	Basal diet +200 mg/kg IsCT

**Table 2:** Basic dietary formulations and nutrient levels (dry matter, %).

Item	1–30 days old	31 ~ 63 days old
Rawmaterial (%)		
Premix^1^	5	5
Corn	55.60	67.30
Soybean meal	32.20	20.50
Bran	2.50	2.50
Fish meal	2.00	2.50
Soybean oil	2.50	2.00
NaCl	0.20	0.20
Total	100.00	100.00
Nutritional level^2^		
Metabolizable energy(MJ/kg)	12.05	12.26
Crude protein	21.60	17.92
Ca	0.89	0.87
Available phosphorus	0.41	0.41
Lys	1.23	0.97
Met	0.50	0.46
Thr	0.80	0.66

^1^This premix provides the following nutrients (per kg of daily feed): Vitamin A: 210 KIU, Vitamin D: 45 KIU, Vitamin E: 550 KIU, Vitamin K3: 40 mg, Vitamin B1: 38 mg, Vitamin B2: 125 mg, Vitamin B6: 75 mg, Vitamin B12: 0.2 mg, Niacin: 1100 mg, Pantothenic Acid: 230 mg, Folic Acid: 18 mg, Biotin: 1 mg, Choline Chloride: 10 mg, Copper: 0.3 mg, Iron: 2.4 mg, Zinc: 1.8 mg, Manganese: 2 mg, Iodine: 16 mg, Selenium: 6.5 mg, Calcium: 14 mg, Phosphorus: 4.6 mg, Methionine: 3 mg, Lysine: 2 mg. ^2^The nutritional levels are calculated values.

### 2.3 Sample collection

The following samples were collected on the 60th day at the end of the study: A 10 mL blood sample was randomly collected from one chicken in each replicate via the brachial vein. The blood was centrifuged at 1,788.8 *g* for 10 min at 4°C, and the serum was separated and stored at −20°C. Four chickens were selected from each replicate, and the thymus, spleen, and bursa of Fabricius were harvested. Caecal contents were collected using cryogenic vials and stored at −80°C.

### 2.4 Growth performance assessment

During the trial period, the feed intake of broilers in each experimental group was recorded by replicates and the average daily feed intake (ADFI) of each group was calculated. The average initial body weight was recorded. On the 60th day of the experiment, the broilers were weighed by replicates. Before weighing, the broilers were fasted for 12 h, but allowed access to water. Average final body weight (AFBW), average daily gain (ADG), and feed-to-gain ratio were calculated for each group.

### 2.5 Blood parameter analysis

Serum was analyzed using an ELISA kit to measure total protein, albumin, globulin, aspartate aminotransferase, alanine aminotransferase (ALT), serum urea nitrogen, glucose, total cholesterol, triglycerides, immunoglobulin A, immunoglobulin M, and immunoglobulin G.

### 2.6 Determination of immune organ indices

The immune organ index was calculated based on the weight of the immune organs, using the following formulae:


$$\text{Liver index}=\text{Liver weight}\;\left(g\right)/\text{Body weight}\;\left(\text{kg}\right)$$

$$\text{Spleen index}=\text{Spleen weight}\;\left(g\right)/\text{Body weight}\;\left(\text{kg}\right)$$

$$\text{Thymus index}=\text{Thymus weight}\;\left(g\right)/\text{Body weight}\;\left(\text{kg}\right)$$

### 2.7 Caecal flora analysis

The collected caecal samples were sent to Shanghai Majorbio Bio-Pharm Technology Co., Ltd. for testing. The raw sequencing data were subjected to quality control, denoising, and chimera removal to obtain valid data. Based on the existing data, OTU clustering, species diversity, and differential abundance analyses were performed. Finally, visualization charts were generated using plotting tools.

### 2.8 Statistical analysis

The data were subjected to rigorous statistical analysis using SPSS software (version 26.0; SPSS Inc., Chicago, IL, United States). The Shapiro–Wilk test was used to assess the normality of the data distribution, and Levene’s test was employed to evaluate the homogeneity of variances for all normally distributed variables. One-way analysis of variance (ANOVA) was performed, followed by a least significant difference (LSD) test for *post hoc* analysis. The significance level for significant differences was set at *p* < 0.05, that for highly significant differences at *p* < 0.01, and that for non-significant differences at *p* > 0.05.

## 3 Results

### 3.1 Effects of antimicrobial peptide IsCT on growth performance of yellow-feathered broilers

Table 3 displays the results of the growth performance measurements. The AFBW and average daily weight gain in the IsCT150 and IsCT200 groups were significantly higher than those in the control (CON) group (*p* < 0.05), with no significant differences between IsCT150 and IsCT200 groups. Compared to the CTC group, the ADFI in the IsCT150 group was significantly lower (*p* < 0.05). The feed conversion ratios in the IsCT150 and IsCT200 groups were significantly lower than those in the IsCT50 and CON groups (*p* < 0.05).

**Table 3:** Effects of IsCT supplementation on growth performance of yellow-feathered broilers.

Item	Group						SE	*p*- value		
	CON	IsCT50	IsCT100	IsCT150	IsCT200	CTC		Overall	Linear	Quadratic
AIBW(g/bird)	30.89	29.98	29.82	30.09	30.02	30.06	0.146	0.365	0.924	0.224
AFBW(g/bird)	2209.62^b^	2239.45	2311.95	2435.96^a^	2422.89^a^	2269.65	30.228	0.126	0.071	0.446
ADFI(g/d)	86.60	86.53	85.49	83.65^b^	85.08	87.75^a^	0.491	0.211	0.060	0.852
ADG(g/d)	34.58^b^	35.07	36.22	38.19^a^	37.98^a^	35.55	0.480	0.125	0.072	0.150
F/G	2.52^a^	2.48^a^	2.37	2.19^c^	2.25^bc^	2.47^ab^	0.038	0.035	0.017	0.475

^a,b,c^Means listed in the same row with different superscripts are significantly different (*p* ≤ 0.05). Diets: CON: control group, corn–soybean-based diet; IsCT50: 50 mg of IsCT was added to each kilogram of diet; IsCT100: 100 mg of IsCT was added to each kilogram of diet; IsCT150: 150 mg of IsCT was added to each kilogram of diet; IsCT200: 200 mg of IsCT was added to each kilogram of diet; CTC: 75 mg of aureomycin was added to the base diet.

### 3.2 Effect of IsCT on immune organ index of yellow-feathered broilers

Table 4 presents the immune organ index measurements. The spleen indices of the IsCT150 and CTC groups were significantly higher than those of the CON and IsCT50 groups (*p* < 0.05). No significant differences were observed in the spleen indices among the IsCT50, IsCT100, and CON groups (*p* > 0.05). The thymus and bursa of Fabricius indices did not differ significantly among the groups (*p* > 0.05).

**Table 4:** Effects of IsCT in diet on immune organ index of yellow-feathered broilers.

Item	Group						SE	*p*- value		
	CON	IsCT50	IsCT100	IsCT150	IsCT200	CTC		Overall	Linear	Quadratic
Thymic index	3.78	3.71	3.83	3.72	3.67	3.69	0.161	1.000	0.875	0.913
Spleen index	2.52^c^	2.53^c^	2.59^bc^	2.99^a^	2.78	2.94^ab^	0.062	0.075	0.542	0.230
Bursa index	1.53	1.85	1.61	1.86	1.62	1.72	0.062	0.616	0.608	0.451

^a,b^Means listed in the same row with different superscripts are significantly different (*p* ≤ 0.05). Diets: CON: control group, corn–soybean-based diet; IsCT50: 50 mg of IsCT was added to each kilogram of diet; IsCT100: 100 mg of IsCT was added to each kilogram of diet; IsCT150: 150 mg of IsCT was added to each kilogram of diet; IsCT200: 200 mg of IsCT was added to each kilogram of diet; CTC: 75 mg of aureomycin was added to the base diet.

### 3.3 Effect of IsCT on serum biochemical indices of yellow-feathered broilers

Table 5 displays the results of the serum biochemical index measurements. Albumin levels in the CTC and IsCT150 groups were significantly higher than those in the CON, IsCT50, and IsCT100 groups. ALT levels in the IsCT150 group were significantly lower than those in the IsCT50, IsCT200, CON, and CTC groups (*p* < 0.05), whereas the CTC group had significantly higher ALT levels than the IsCT groups.

**Table 5:** Effects of IsCT in diet on serum biochemical indices of yellow-feathered broilers.

Item	Group						SE	*p*- value		
	CON	IsCT50	IsCT100	IsCT150	IsCT200	CTC		Overall	Linear	Quadratic
TP(g/L)	28.70	33.70	37.70	29.90	31.40	35.00	1.304	0.398	0.857	0.077
ALB(g/L)	11.57^c^	11.67^c^	12.55^bc^	13.84^ab^	13.09	14.53^a^	0.360	0.034	0.680	0.056
GLB(g/L)	16.65	21.55	23.80	18.55	21.20	21.45	0.970	0.382	0.710	0.141
AST(U/L)	206.50	179.50	188.50	169.00	200.50	196.50	5.682	0.481	0.529	0.699
ALT(U/L)	3.00^ab^	2.50^bc^	2.50^bc^	1.05^c^	1.50^bc^	4.00^a^	0.331	0.059	0.023	0.063
GLU(mmol/L)	15.50	11.75	10.55	12.90	11.95	12.20	0.665	0.450	0.555	0.207

^a,b,c^Means listed in the same row with different superscripts are significantly different (*p* ≤ 0.05). Diets: CON: control group, corn–soybean-based diet; IsCT50: 50 mg of IsCT was added to each kilogram of diet; IsCT100: 100 mg of IsCT was added to each kilogram of diet; IsCT150: 150 mg of IsCT was added to each kilogram of diet; IsCT200: 200 mg of IsCT was added to each kilogram of diet; CTC: 75 mg of aureomycin was added to the base diet.

### 3.4 Effect of IsCT on serum immune protein of yellow-feathered broilers

Table 6 displays the results of serum immune protein measurements. IgA levels in the IsCT150, IsCT200, and CTC groups were significantly higher than those in the IsCT50 group (*p* < 0.05). IgG levels in the CTC, IsCT100, IsCT150, and IsCT200 groups were significantly higher than those in the IsCT50 and CON groups (*p* < 0.05). IgM levels in the IsCT150, IsCT200, and CTC groups were significantly higher than those in the IsCT50 and CON groups (*p* < 0.05).

**Table 6:** Effects of IsCT in diet on serum immune protein of yellow-feathered broilers.

Item	Group						SE	*p*- value		
	CON	IsCT50	IsCT100	IsCT150	IsCT200	CTC		Overall	Linear	Quadratic
IgA(μg/mL)	357.98	343.57^b^	391.61	400.73^a^	403.39^a^	411.49^a^	8.063	0.070	0.106	0.283
IgG(μg/mL)	2715.10^b^	2699.67^b^	3082.42^a^	3100.58^a^	3053.07^a^	3103.30^a^	51.342	0.020	0.021	0.719
IgM(μg/mL)	986.76^b^	975.21^b^	1051.95	1102.62^a^	1093.72^a^	1110.14^a^	16.130	0.030	0.121	0.224

^a,b^Means listed in the same row with different superscripts are significantly different (*p* ≤ 0.05). Diets: CON: control group, corn–soybean-based diet; IsCT50: 50 mg of IsCT was added to each kilogram of diet; IsCT100: 100 mg of IsCT was added to each kilogram of diet; IsCT150: 150 mg of IsCT was added to each kilogram of diet; IsCT200: 200 mg of IsCT was added to each kilogram of diet; CTC: 75 mg of aureomycin was added to the base diet.

### 3.5 Effect of dietary IsCT antimicrobial peptide on caecal flora alpha diversity in yellow-feathered broilers

#### 3.5.1 Alpha diversity of fecal bacteria

Table 7 displays the results of the *α*-diversity indices. The Shannon index of the CON group was significantly higher than that of the CTC group (*p* < 0.05). The Simpson index of the CTC group was significantly higher than that of the other groups (*p* < 0.05). Compared with the CTC group, the Abundance-based Coverage Estimator (ACE) indices of both the CON and IsCT groups were significantly higher (*p* < 0.05). Furthermore, the good coverage index of the CTC group was significantly higher than that of the CON group (*p* < 0.01).

**Table 7:** Determination of cecal microbiota α-diversity results among different treatment groups.

Item	Group						SE	*p*- value		
	CON	IsCT50	IsCT100	IsCT150	IsCT200	CTC		Overall	Linear	Quadratic
Chao1 index	923.91	852.16	802.23	748.80	874.25	650.83	30.395	0.103	0.026	0.927
Shannon index	4.17^Aa^	4.03^a^	4.24	4.00	4.41^a^	3.68^Bb^	0.084	0.182	0.345	0.244
Simpson index	0.05	0.06	0.04^b^	0.05	0.04^b^	0.09^a^	0.006	0.221	0.231	0.109
Ace index	963.15^a^	880.68	835.23	773.77	907.10^a^	675.41^b^	33.326	0.146	0.035	0.964
good_coverage index	0.9936^B^	0.9944	0.9946	0.9949	0.9947	0.9959^A^	0.000	0.152	0.014	0.922

^a,b^Means listed in the same row with different superscripts are significantly different (*p* ≤ 0.05). ^A,B^Means listed in the same row with different superscripts are highly significantly different (*p* ≤ 0.01). Diets: CON: control group, corn–soybean-based diet; IsCT50: 50 mg of IsCT was added to each kilogram of diet; IsCT100: 100 mg of IsCT was added to each kilogram of diet; IsCT150: 150 mg of IsCT was added to each kilogram of diet; IsCT200: 200 mg of IsCT was added to each kilogram of diet; CTC: 75 mg of aureomycin was added to the base diet.

### 3.6 Effects of dietary IsCT on beta diversity of caecal microflora in yellow-feathered broilers

**Figure 1:**
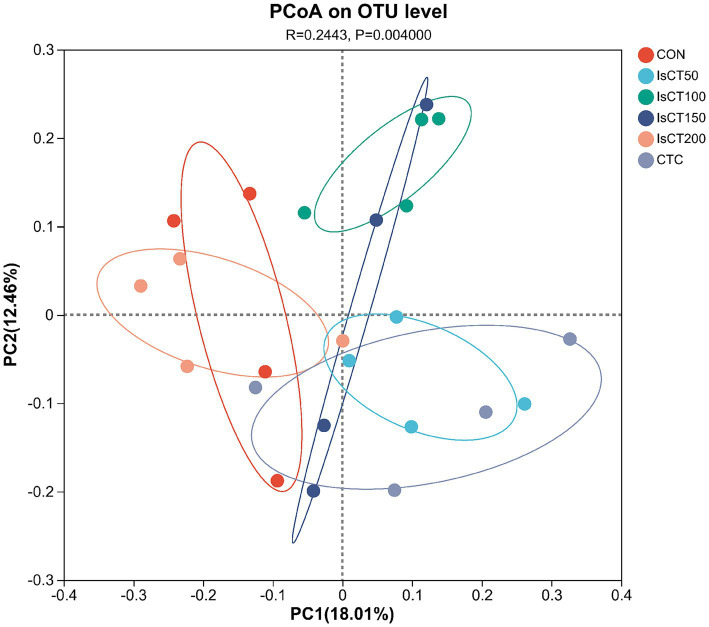
Principal component and principal coordinate analyses of fecal bacteria (results based on PCOA weighted principal coordinate analysis). Diets: CON: control group, corn–soybean-based diet; IsCT50: 50 mg of IsCT was added to each kilogram of diet; IsCT100: 100 mg of IsCT was added to each kilogram of diet; IsCT150: 150 mg of IsCT was added to each kilogram of diet; IsCT200: 200 mg of IsCT was added to each kilogram of diet; CTC: 75 mg of aureomycin was added to the base diet.

Principal coordinate analysis (PCoA) was used to visualize the microbial community structure and compare differences among experimental groups. In Figure 1, each point represents a sample and different colors indicate different groups. The first and second principal coordinates, PC1 and PC2, accounted for 18.01 and 12.46% of the variance in caecal microbial structure, respectively. Samples from the IsCT50, IsCT100, IsCT150, and CTC groups were distinctly separated from those of the CON group, indicating significant differences in bacterial communities compared to the CON group. In contrast, the samples from the CON group clustered together with those from the IsCT200 group, showing no significant differences.

### 3.7 Effects of dietary IsCT on beta diversity of caecal microflora in yellow-feathered broilers

**Figure 2:**
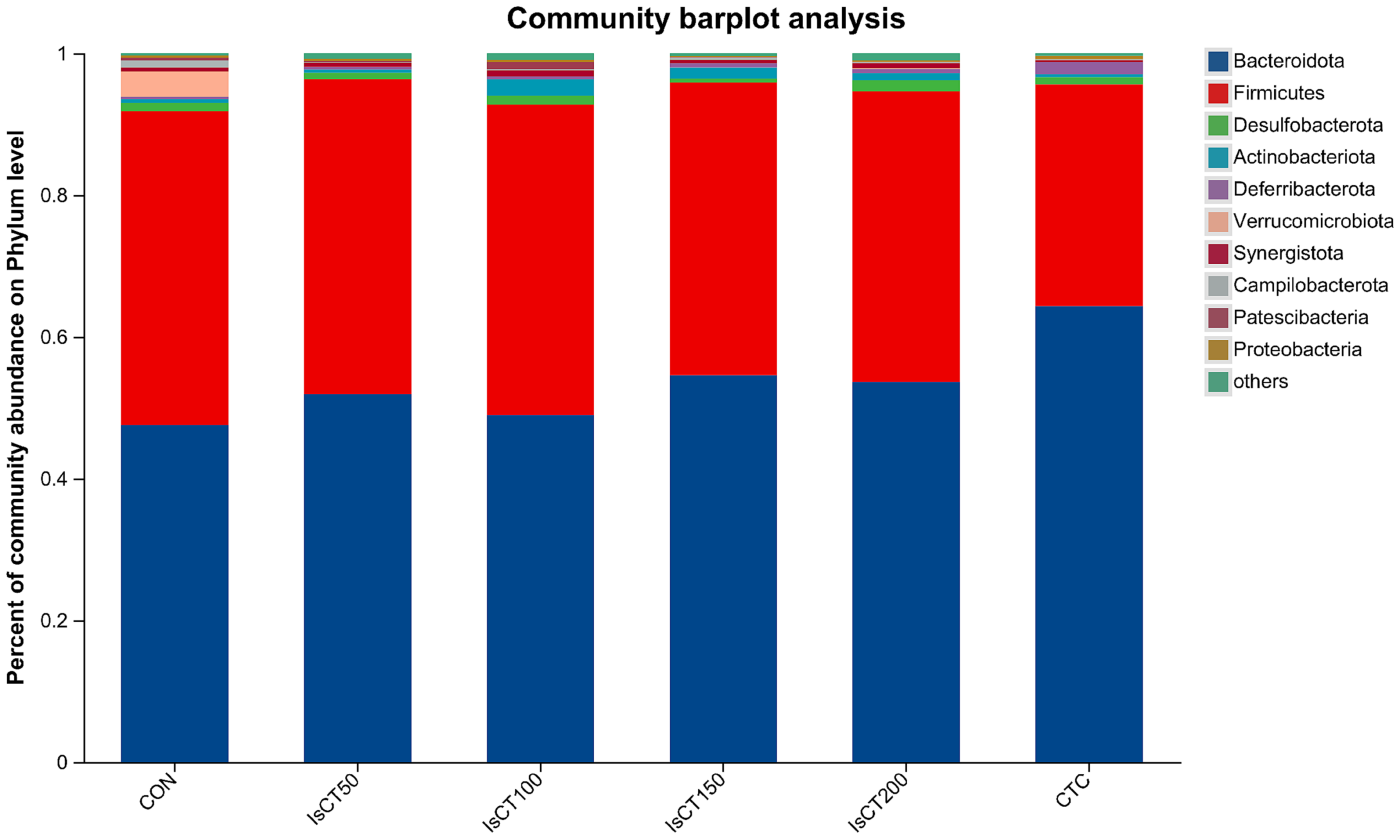
The relative abundance of microbial species at the phylum level in the cecum of yellow-feathered broilers under different treatments. Diets: CON: control group, corn–soybean-based diet; IsCT50: 50 mg of IsCT was added to each kilogram of diet; IsCT100: 100 mg of IsCT was added to each kilogram of diet; IsCT150: 150 mg of IsCT was added to each kilogram of diet; IsCT200: 200 mg of IsCT was added to each kilogram of diet; CTC: 75 mg of aureomycin was added to the base diet.

**Figure 3:**
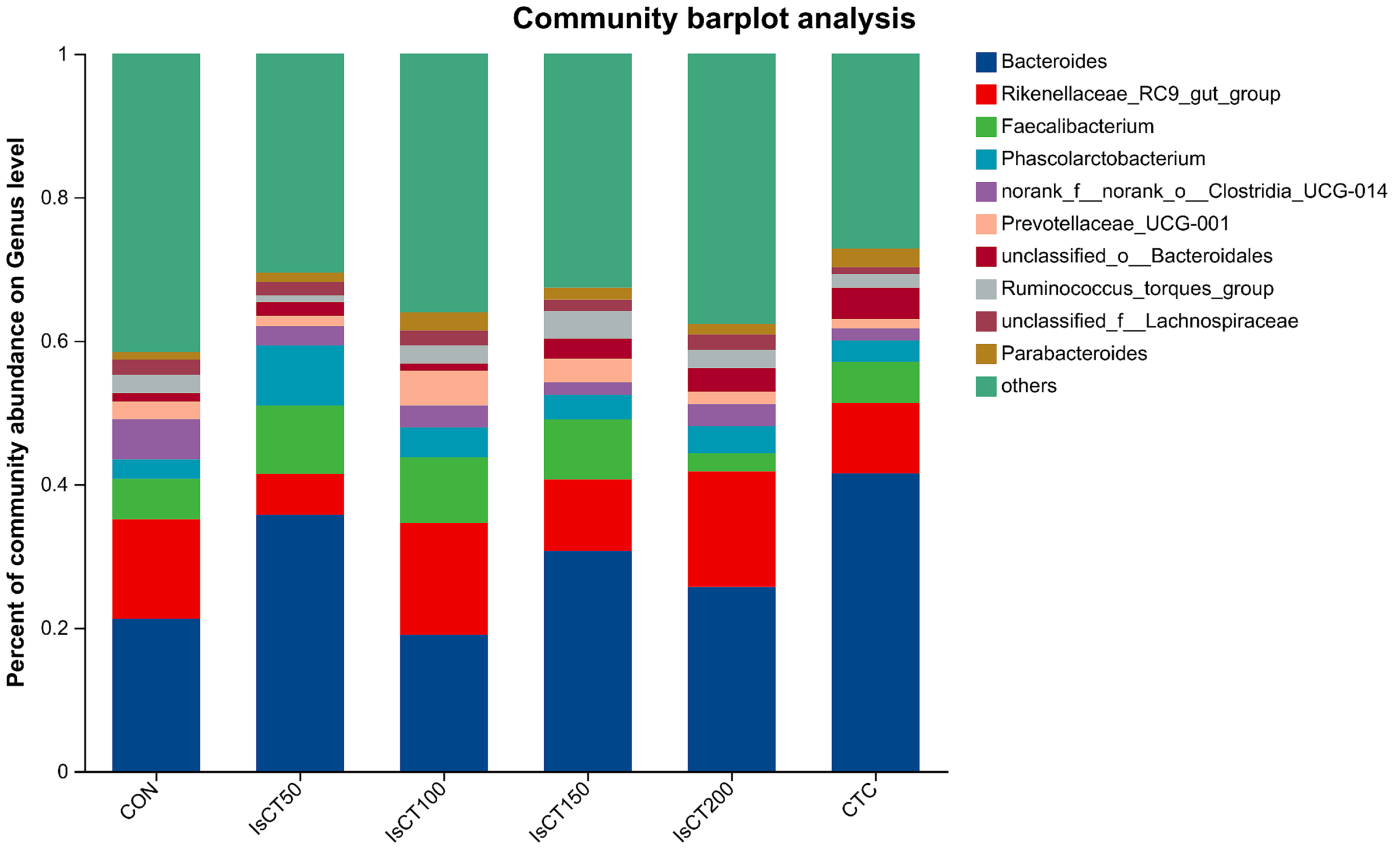
The relative abundance of microbial species at the genus level in the cecum of yellow-feathered broilers under different treatments. Diets: CON: control group, corn–soybean-based diet; IsCT50: 50 mg of IsCT was added to each kilogram of diet; IsCT100: 100 mg of IsCT was added to each kilogram of diet; IsCT150: 150 mg of IsCT was added to each kilogram of diet; IsCT200: 200 mg of IsCT was added to each kilogram of diet; CTC: 75 mg of aureomycin was added to the base diet.

The main microbial phyla present in the caecal microbiota of yellow-feathered broilers at 60 days of age included Bacteroidetes, Firmicutes, Desulfobacterota, Actinobacteriota, and Deferribacterota (Figure 2), while the main microbial genera included Bacteroides, Rikenellaceae_RC9_gut_group, Faecalibacterium, Phascolarctobacterium, and the norank_f__norank_o__Clostridia_UCG-014 (Figure 3).

### 3.8 Analysis of species diversity in the intestinal microbiota

**Figure 4:**
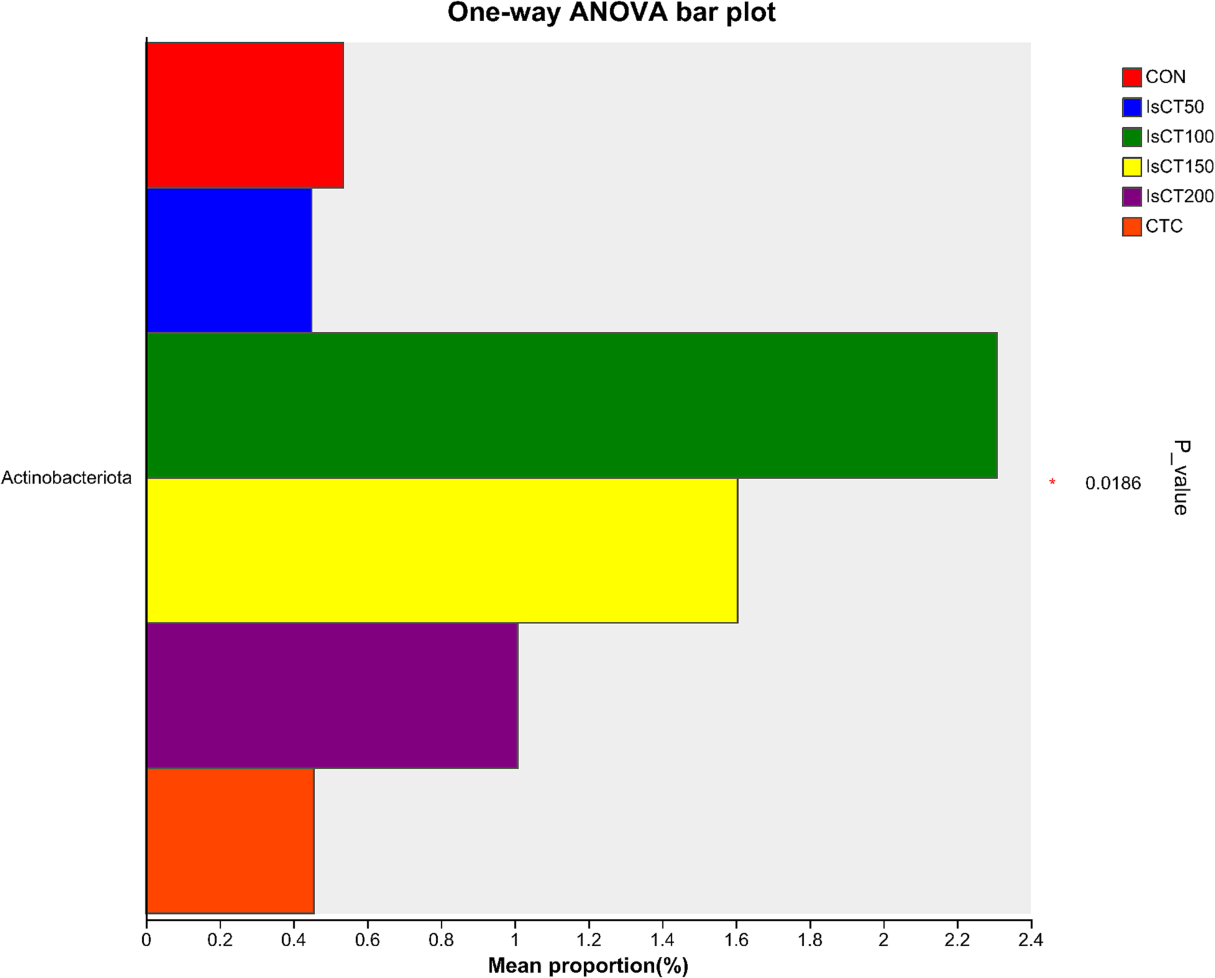
T-test of cecal microbiota at the phylum level in yellow-feathered broilers under different treatments. Diets: CON: control group, corn–soybean-based diet; IsCT50: 50 mg of IsCT was added to each kilogram of diet; IsCT100: 100 mg of IsCT was added to each kilogram of diet; IsCT150: 150 mg of IsCT was added to each kilogram of diet; IsCT200: 200 mg of IsCT was added to each kilogram of diet; CTC: 75 mg of aureomycin was added to the base diet.

**Figure 5:**
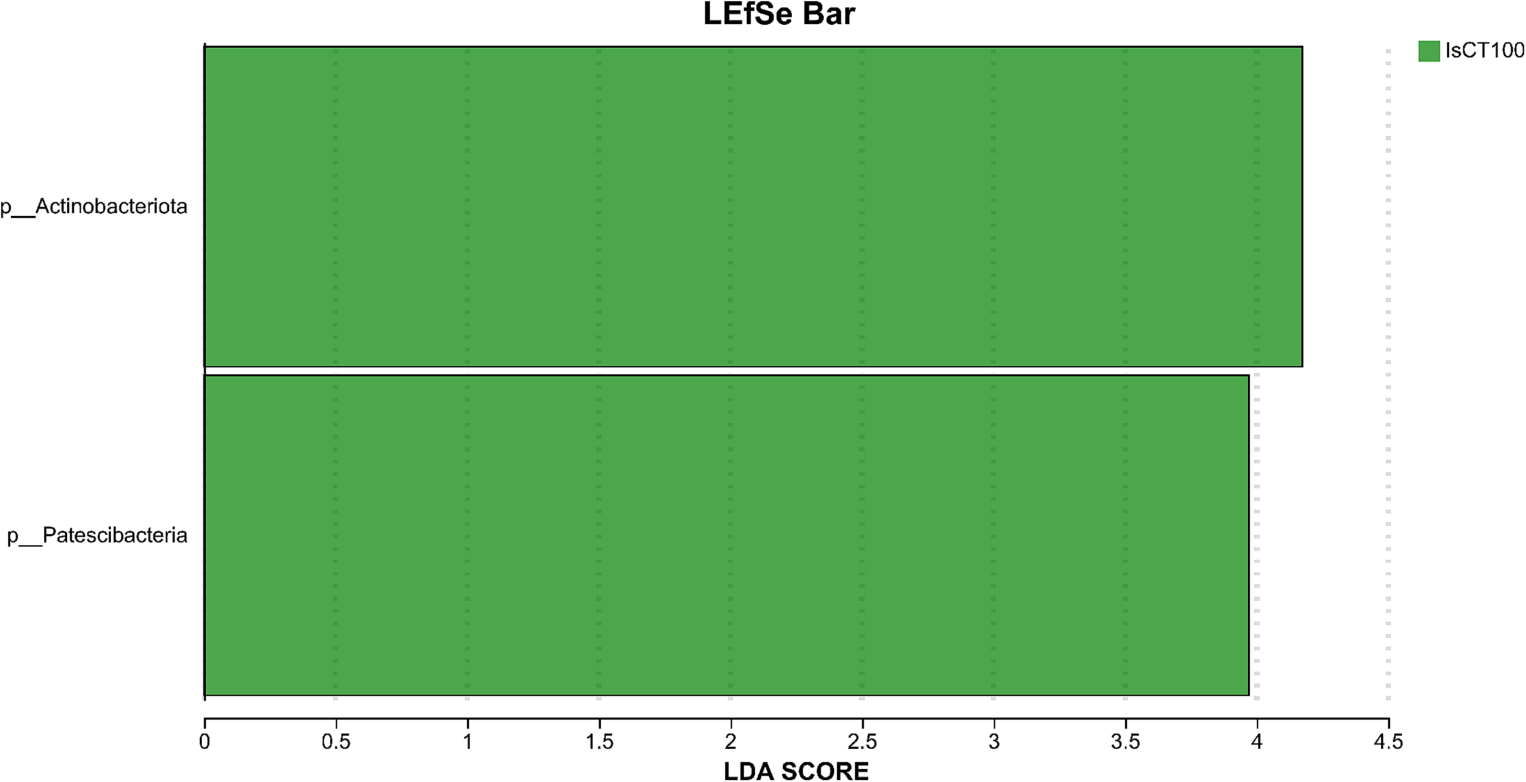
The relative species differences of the microbiota at the phylum level in the cecum of yellow-feathered broilers under different treatments. Diets: CON: control group, corn–soybean-based diet; IsCT50: 50 mg of IsCT was added to each kilogram of diet; IsCT100: 100 mg of IsCT was added to each kilogram of diet; IsCT150: 150 mg of IsCT was added to each kilogram of diet; IsCT200: 200 mg of IsCT was added to each kilogram of diet; CTC: 75 mg of aureomycin was added to the base diet.

**Figure 6:**
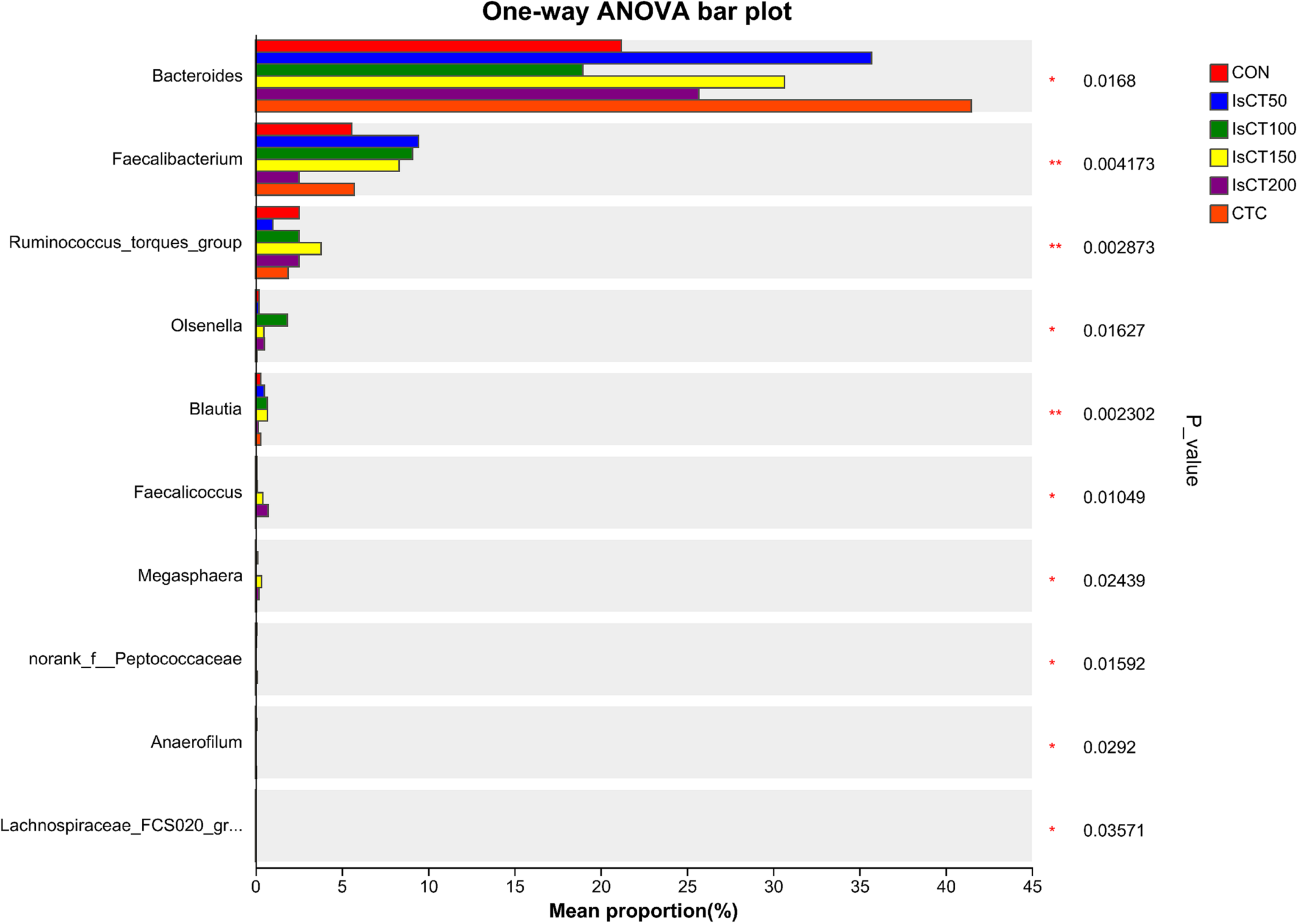
T-test of cecal microbiota at the genus level in yellow-feathered broilers under different treatments. Diets: CON: control group, corn–soybean-based diet; IsCT50: 50 mg of IsCT was added to each kilogram of diet; IsCT100: 100 mg of IsCT was added to each kilogram of diet; IsCT150: 150 mg of IsCT was added to each kilogram of diet; IsCT200: 200 mg of IsCT was added to each kilogram of diet; CTC: 75 mg of aureomycin was added to the base diet.

**Figure 7:**
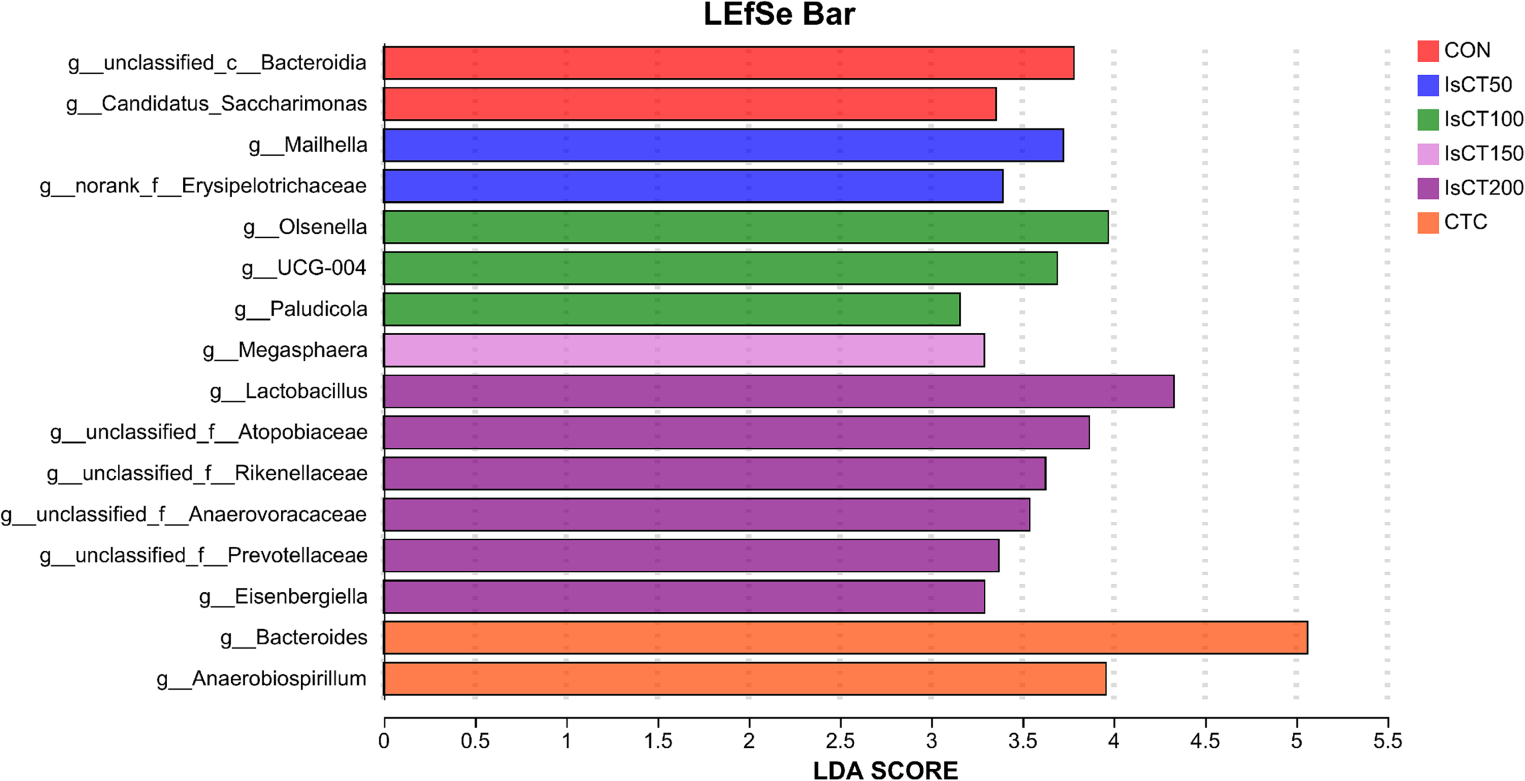
The relative species differences of the microbiota at the genus level in the cecum of yellow-feathered broilers under different treatments. Diets: CON: control group, corn–soybean-based diet; IsCT50: 50 mg of IsCT was added to each kilogram of diet; IsCT100: 100 mg of IsCT was added to each kilogram of diet; IsCT150: 150 mg of IsCT was added to each kilogram of diet; IsCT200: 200 mg of IsCT was added to each kilogram of diet; CTC: 75 mg of aureomycin was added to the base diet.

Linear discriminant analysis (LDA) was used to determine the differences in microbial composition at the phylum and genus levels in the caecum among different experimental groups (LDA score ≥ 3.5). At the phylum level (Figures 4, 5), the relative abundances of Actinobacteriota (p__Actinobacteriota) and Patescibacteria (p__Patescibacteria) in the IsCT100 group were significantly higher than those in the other groups (*p* < 0.05). At the genus level (Figures 6, 7), the relative abundance of unclassified genera within the class Bacteroidia (g__unclassified_c__Bacteroidia) and that of Candidatus Saccharimonas (g__Candidatus_Saccharimonas) in the CON group was significantly higher than that in the other groups (*p* < 0.05). In the IsCT50 group, the relative abundances of Mailhella (g__Mailhella) and unclassified genera within the family Erysipelotrichaceae (g__norank_f__Erysipelotrichaceae) significantly increased (*p* < 0.05). In the IsCT100 group, the relative abundances of Olsenella (g__Olsenella), unclassified genus UCG-004 (g__UCG-004), and Paludicola (g__*Paludicola*) were significantly higher. In the IsCT150 group, the relative abundance of Megasphaera (g _ *Megasphaera*) significantly increased (*p* < 0.05). In the IsCT treatment groups, the relative abundances of Lactobacillus (g _ *Lactobacillus*), *Eisenbergiella* (g _ *Eisenbergiella*), Sphaerochaeta (g _ *Sphaerochaeta*), and several unclassified genera (e.g., g _ unclassified_f _ *Atopobiaceae*) were significantly higher (*p* < 0.05). In the CTC group, the relative abundances of Bacteroides (g__*Bacteroides*) and *Anaerobiospirillum* (g__*Anaerobiospirillum*) was significantly higher than in the other groups (*p* < 0.05).

## 4 Discussion

The antimicrobial peptide Plectasin improves the growth performance of broiler chickens, which may be mediated by the inhibition of harmful bacteria in the intestine (14). Another study found that Microcin C7 increased the ADG of broilers and reduced their feed-to-gain ratio (31). Additionally, research indicated that AMPs promoted broiler growth (32). This study found that adding 150 and 200 mg/kg IsCT to the diet significantly increased the final body weight and ADG of yellow-feathered broilers at 60 days of age. Moreover, 150 mg/kg IsCT significantly reduced the ADFI of yellow-feathered broilers. These findings suggest that supplementing broiler diets with IsCT can improve growth performance, possibly by enhancing intestinal barrier function and immune performance, thereby improving nutrient digestion and absorption. Similar additives include essential oils, probiotics and polysaccharide. These additives improve gut health, thereby mediating enhanced growth performance (33–35). What differs in certain aspects is that IsCT possesses a small molecular weight (only 13 amino acids) and a simple composition, allowing it to be more easily metabolized without leaving residues in the animal’s body.

Immune organ indicators are commonly used to evaluate the immune performance of poultry (36), and an increase in the immune organ index often indicates good immune organ development (37). The spleen is involved in both humoral and cellular immune responses, making it an important immune organ in poultry (38). Studies have found that adding Mastoparan X to the diet of bacteria-challenged chickens can increase their relative spleen weight (23). Another study indicated that feeding broilers the antimicrobial peptide Gal-13 significantly increased the weight of their spleens and thymuses (39). This study found that adding 150 mg/kg IsCT to the diet significantly increased the spleen index of yellow-feathered broilers, which is consistent with previous findings, indicating that IsCT has a positive effect on spleen development in broilers.

According to previous studies, the addition of antimicrobial peptides to feed can effectively improve animal serum indicators (40, 41). Studies have found that adding bioactive peptides from sesame meal to the diet of broilers can increase the concentration of serum albumin (42), which is consistent with the results of this study. Another study indicated that supplementing the diet with the antimicrobial peptide LLv raised serum IgA and IgM levels in broilers at 21 and 42 days of age (43). Additionally, it has been found that antimicrobial peptides increased serum IgA and IgG levels in Gushi chickens (44). In this study, IsCT significantly increased serum IgA, IgM, and IgG levels, which is consistent with previous literature. ALT is not only closely related to liver fat accumulation (45, 46) but also plays an important role in the intermediate metabolism of amino acids and glucose (47). ALT is closely associated with metabolic health in animals and serves as a marker of liver disease (48). Some studies have shown that dietary lysozymes and ZnB significantly reduce ALT levels (49). In this study, we found that IsCT reduced serum ALT content in broilers, indicating an improvement in liver function. Additionally, ALT levels increased significantly in broilers fed with antibiotics. Research has shown that long-term use of antibiotics can have adverse effects on the liver, including significant increases in ALT levels (50–52). Based on previous studies, we speculated that the elevated serum ALT levels in the antibiotic group of broilers in this study were caused by long-term antibiotic feeding.

The gut microbiota is a key contributor to maintaining intestinal homeostasis and host health, and has been shown to play a role in nutrient absorption, metabolism, immune system maturation, and the intestinal mucosal barrier (53, 54). Indicators reflect the richness and evenness of species within a single sample (55). Studies have shown that the Chicken NK-2 peptide effectively reduces the reproductive capacity of *Eimeria* parasites in the host’s intestines (14). Another study showed that the antimicrobial peptide cLFchimera restored the microbial community balance in the ileum of birds disrupted by antibiotics (16). Through *α*-diversity analysis, it was found that IsCT reduced the richness of the caecal microbiota in broilers, which may be due to the inhibitory effect of IsCT on certain genera. *β*-diversity is an important indicator of differences in species composition between different samples (56). Through β-diversity analysis, it was found that the composition of the microbiota in the IsCT group differed significantly from that in the CON group, suggesting that the antimicrobial peptide IsCT had some impact on the caecal microbiota structure of broilers. In the caecum of broilers, *Bacteroidetes* and *Firmicutes* are the two major bacterial phyla. They play important roles in the gut microbial community, and are closely associated with host nutrient absorption, immune regulation, and health status. Intestinal-associated Bacteroidetes can degrade complex polymers, thereby facilitating food digestion and nutrient acquisition (57). The study found that at the phylum level, the abundance of *Bacteroidetes* in the caecal microbiota of broilers increased, whereas the abundance of *Firmicutes* decreased, which may improve the intestinal immune status, enhance mucosal health, and potentially reduce excessive energy absorption, thereby positively impacting the health and growth performance of broilers (58, 59). Notably, some species of the genus Bacteroides secrete short-chain fatty acids that can influence gut and brain functions (60). The metabolites produced by these microorganisms may induce autism spectrum disorder (61). However, the molecular mechanisms by which Bacteroidetes affect the host remain to be explored. Additionally, LDA analysis showed that at the phylum level, the relative abundances of *Actinobacteriota* and *Patescibacteria* were significantly higher in the IsCT100 group than in the other groups. Although *Actinobacteriota* constitute a relatively small proportion of the gut microbiota, they play a crucial role in maintaining intestinal homeostasis (62). *Patescibacteria* typically coexist symbiotically with other microbes, with certain *Patescibacteria* forming mutualistic relationships with *Actinobacteria*, suggesting that they play a special ecological role in the gut (63).

## 5 Conclusion

This study concluded that adding IsCT to the diet of yellow-feathered broiler chickens could improve their growth performance, immune function, and gut microbiota, with an optimal supplementation level of 150 mg/kg.

## 6 Simple summary

We aimed to investigate the effects of Isalo scorpion cytotoxic peptide (IsCT) on the growth, immune performance, and intestinal microbiota of yellow-feathered broilers. A total of 240 yellow-feathered broilers were randomly assigned to six groups, with different doses of IsCT added to the basal diet of each group. The results showed an increase in immunoglobulin levels and daily weight gain and reduction in daily feed intake and feed conversion ratio. Additionally, the abundance of beneficial bacteria increased whereas that of harmful bacteria decreased in the caecum of the chickens. Therefore, IsCT helps improve the growth and immune performance of yellow-feathered broilers and optimizes the intestinal microbiota.

## Data Availability

The data presented in the study are deposited in the NCBI bioproject for the 16S sequencing data repository, accession number PRJNA1194524.
